# Species mtDNA genetic diversity explained by infrapopulation size in a host‐symbiont system

**DOI:** 10.1002/ece3.1842

**Published:** 2015-11-24

**Authors:** Jorge Doña, Marina Moreno‐García, Charles D. Criscione, David Serrano, Roger Jovani

**Affiliations:** ^1^ Department of Evolutionary EcologyEstación Biológica de Doñana (CSIC)Avda. Americo Vespucio s/nSevillaSpain; ^2^ Department of BiologyTexas A&M University3258 TAMUCollege StationTexas77843; ^3^ Department of Conservation BiologyEstación Biológica de Doñana (CSIC)AvdaAmerico Vespucio s/nSevillaSpain

**Keywords:** COI, demography, feather mites, genetic diversity, host‐parasite interactions, mtDNA

## Abstract

Understanding what shapes variation in genetic diversity among species remains a major challenge in evolutionary ecology, and it has been seldom studied in parasites and other host‐symbiont systems. Here, we studied mtDNA variation in a host‐symbiont non‐model system: 418 individual feather mites from 17 feather mite species living on 17 different passerine bird species. We explored how a surrogate of census size, the median infrapopulation size (i.e., the median number of individual parasites per infected host individual), explains mtDNA genetic diversity. Feather mite species genetic diversity was positively correlated with mean infrapopulation size, explaining 34% of the variation. As expected from the biology of feather mites, we found bottleneck signatures for most of the species studied but, in particular, three species presented extremely low mtDNA diversity values given their infrapopulation size. Their star‐like haplotype networks (in contrast with more reticulated networks for the other species) suggested that their low genetic diversity was the consequence of severe bottlenecks or selective sweeps. Our study shows for the first time that mtDNA diversity can be explained by infrapopulation sizes, and suggests that departures from this relationship could be informative of underlying ecological and evolutionary processes.

## Introduction

Understanding what shapes among‐species variation in genetic diversity remains a major challenge in evolutionary ecology (Leffler et al. 2012; Romiguier et al. 2014; Fujisawa et al. 2015). This is especially true for parasites (Criscione et al. 2005; Huyse et al. 2005; Poulin 2011), despite being a widespread lifestyle (Poulin 2011) and a key element of ecosystems (Lafferty et al. 2006). Knowledge of what impacts parasite genetic diversity also has implications for host health and conservation (Criscione et al. 2005; Whiteman and Parker 2005).

Parasites and other symbionts (including commensals and mutualists) live in patchy and ephemeral habitats (i.e., individual hosts) and Price (1980) predicted that parasites, in contrast with free‐living species, would have a lower genetic diversity. However, we currently know that parasite genetic diversity is comparable to that of free‐living organisms and variable between parasite species (Nadler 1990; Bush et al. 1997, 2001; Blouin et al. 1999; Criscione and Blouin 2005; Poulin 2011). The question that remains then is what dictates the variation in genetic diversity among parasites species and other symbionts in host‐symbiont interactions.

Population genetic theory predicts that neutral genetic diversity should increases proportionally with the effective population size, *N*_*e*_ (i.e., the size of a Wright‐Fisher ideal population that experiences the same rate of drift as the population under consideration). In fact, recent correlative studies that used surrogates of census population sizes of various free‐living species have found support for this prediction (McCusker and Bentzen 2010; Romiguier et al. 2014; Fujisawa et al. 2015). For parasites, several factors such as aggregated distributions, crowding effects, and host immunity could influence the *N*_*e*_ of parasite component populations (i.e., all the parasites among all the infected hosts within the host population; Bush et al. 1997) (Criscione and Blouin 2005). As an initial null expectation, Criscione et al. (2005) hypothesized that parasite species with larger census sizes would have larger *N*_*e*_, and thus higher genetic diversity. Interestingly, Criscione et al. (2005) found tentative support for this hypothesis by qualitatively correlating published mtDNA diversity data for seven nematode species and their mean intensities of infection, i.e., the mean number of parasites per infected host. Their prediction is based on the assumption that the census size of the parasite component population is reflected by the mean infrapopulation size (i.e., mean infection intensities) (Criscione et al. 2005). Mean intensities were used because an estimate of the census component population would require an estimate of the host population size as well. To our knowledge, there has yet to be an explicit statistical test to determine if infection intensities correlate with genetic diversity in parasites.

**Figure 1:**
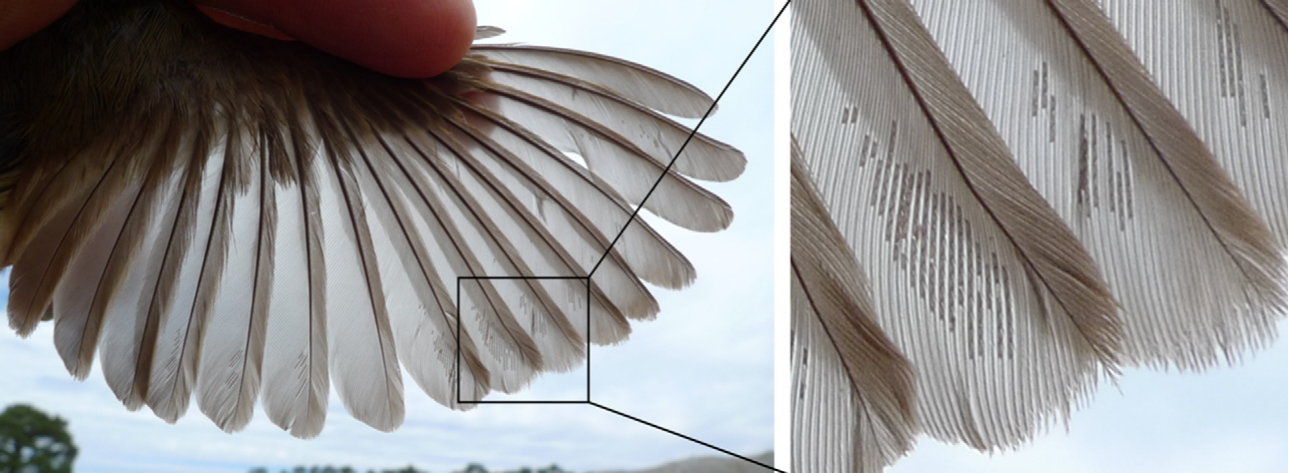
*Dolichodectes hispanicus* feather mites on the wing flight feathers of a Melodious warbler *Hippolais polyglotta*. Infrapopulation size is measured by counting the number of mites exposing the wing against daylight.

Here, we test the hypothesis that species‐wide mitochondrial (mtDNA) genetic diversity in a group of ectosymbiont species is correlated with their median infrapopulation sizes. The finding of a significant relationship would indicate that infrapopulation sizes influence species‐wide *N*_*e*_ (or more specifically the effective sizes of the mtDNA of each species). We test this hypothesis using 17 feather mite species living on 17 different passerine bird species. Feather mites (Acari: Astigmata: Analgoidea and Pterolichoidea) are among the most abundant ectosymbionts living on birds (Gaud and Atyeo 1996; Proctor 2003). Feather mite median intensity strongly and consistently differs among bird species when studied in different populations and habitats (Díaz‐Real et al. 2014), so median infrapopulation size can be considered as an inherent characteristic of each host species. Within feather mites some families are parasites of the skin and nasal cavities of birds. However, most species are highly specialized to different plumage microhabitats (plumicolous mites, hereafter feather mites), where they cause no visible damage to their hosts (Blanco et al. 2001; Dowling et al. 2001; Galván et al. 2008; Fig. 1). Recent studies even suggest that these feather mites may be commensals or even mutualists of birds, feeding on keratinophilic fungi and feather degrading bacteria (Blanco et al. 2001; Galván et al. 2012). Nonetheless, they share commonalities to the parasite life style in that feather mites expend their whole life on their bird hosts, mainly transmitting from bird parents to offspring on the host nest (Dubinin 1951; Dabert and Mironov 1999; Proctor 2003; Dabert et al. 2015).

## Materials and Methods

### Data

418 individual mites from 17 bird species (one mite species for each bird species) were sampled by capturing birds with mist nets and collecting all present mites from the wing flight feathers using a cotton swab impregnated with ethanol and preserved at −20°C in tubes with 96% ethanol for posterior lab separation. Sampling was done in two steps (Table S1, Supporting information).

First, in different localities of South Spain during 2014, we chose (see stereomicroscope separation below) five mite individuals from each of two to five individual birds from each of 14 bird species (one mite species per bird species). This sample consisted of a total of 297 individual mites. Sampling details (i.e., locality, date of sampling, and sample size) can be found in Table S1 (Supplementary information). From previous taxonomic (Atyeo and Braasch 1966; Gaud and Atyeo 1996) and molecular (Doña et al. 2015) information, we had a reasonable knowledge on the common feather mite species living on our study bird species. Thus, once in the laboratory, we identified mite specimens corresponding to the most abundant feather mite species living on each bird species under a stereomicroscope according to basic morphological traits. Subsequent molecular barcoding identification (Doña et al. 2015) confirmed initial identifications in all cases (see also Díaz‐Real et al. 2015).

Second, we included 91 mtDNA sequences (91 mite individuals) from the same 14 mite species, coming from Doña et al. (2015), where we sampled from 2011 to 2013 in Spain and Russia one mite individual from each bird individual (Table S1, Supporting information). Moreover, 30 sequences from three new mite species were included from Doña et al. (2015) (Table S1, Supporting information). [Other mite species with less than nine sequences in Doña et al. (2015) were not included to be conservative with the minimum sample size suggested in Goodall‐Copestake et al. (2012)].

In addition, we included 34 individual mites (i.e., 25 new samples from the field and 9 from Doña et al. (2015)) from *Proctophyllodes serini*, a species we a priori did not expect to fulfill our prediction due to its recent evolutionary history (see Discussion). In particular, we were interested on exploring whether the recent divergence of this species translated into a departure from the general relationship between infrapopulation size and genetic diversity. Therefore, we conducted two analyses: one that included *P. serini* (18 species total) and one that did not (17 species total).

Median intensity significantly differs among feather mite species and is moderately repeatable (Díaz‐Real et al. 2014). Here, median infrapopulation size for each mite species was obtained from Díaz‐Real et al. (2014), whose data set is the largest so far available for feather mite loads in European passerine birds (gathered by counting the number of mites exposing the wing against daylight; Fig. 1). For the bird species analysed here, this data set includes a total of 6,607 individual birds, with a mean (range) of 389 (11‐2,197) birds per species.

### DNA analysis

Genomic DNA was extracted using HotSHOT (Truett et al. 2000). A segment of approximately 650 bp of the COI region was amplified. This marker has been shown to be useful to characterize the genetic diversity in other species (Delrieu‐Trottin et al. 2014; Fujisawa et al. 2015). In addition, this marker offers within‐species polymorphism in feather mites species (Doña et al. 2015). PCRs were done by using degenerate primers bcdF05 (50‐TTTTCTACH AAYCATAAAGATATTGC‐30) and bcdR04 (50‐TATAAACYTCDGGATGNCCAA AAAA‐30) (Dabert et al. 2008). PCRs were carried out in 20 *μ*L reaction volumes containing 1x (NH_4_)_2_SO_4_ reaction buffer (Bioline), 2.5 mM MgCl2, 19 BSA, 0.25 mM DNTPs, 2 *μ*m of each primer, 1.25 U BIOTAQTM (Bioline), and 7 *μ*L of DNA template. The reaction followed a touchdown PCR profile: 95°C for 3 min, 20 cycles of 95°C for 1 min, 55°C for 30 s with a decrease of 0.5°C every cycle, 72°C for 1 min, and 20 cycles of 95°C for 1 min, 45°C for 30 s and 72°C for 1 min, with a final extension step of 72°C for 5 min. PCR products were quantitatively assessed by electrophoresis on a 2% agarose gel, and visible bands corresponding to the COI fragment size were sequenced in both directions. COI sequencing was carried out using the Sanger method and performed by Macrogen, Europe (Holland).

Sequences, including those from Doña et al. (2015), were visually edited and manually trimmed to 507 bp using Geneious v4.7 (Drummond et al. 2009). New sequences were deposited in GenBank with the accession nos. KT025257‐ KT025578. Sequences were aligned using MUSCLE with default settings (Edgar 2004). Clustering of species was evaluated on Neighbor‐joining trees done in MEGA 6 (Tamura et al. 2013).

### Data analysis

#### Haplotype networks, nucleotide diversity, and repeatability of nucleotide diversity

We used the *haplotype* and *haplonet* functions from the PEGAS R package to construct haplotype networks (Paradis 2010). Nucleotide diversity (*π*, hereafter genetic diversity; Nei 1987) was calculated using the *nuc.div* function implemented in the R package PEGAS (Paradis 2010). This function computes the sum of the number of differences between pairs of sequences divided by the number of comparisons.

Our study is based on the assumption that our sampling was good enough to obtain a reasonable estimate of the overall genetic diversity for each mite species. This assumption is reasonably supported by current evidence. First, no genetic structure has been found in any species of feather mite studied to date in relation to the migratory status of the host species (Fernández et al. 2013), or in relation to the sampling locality in our previous study from distant geographic localities (Russia vs. Spain, Doña et al. 2015). Moreover, a visual inspection of haplotype networks of the sequences analyzed here showed no evidence of geographic structure (Figs. S3–S12 Supporting information), and no consistent difference was found within species in genetic diversity estimates when including or not the 42 Russian samples (paired *t* test *t *= *−*0.245, df=31.994, *P* = 0.8; Fig. S13, Supporting information).

Second, we used the *ICCbare* function of the ICC R package (Wolak et al. 2012) to calculate the intraclass correlation coefficient (i.e., repeatability, *R*), that is, to test how repeatable is genetic diversity among infrapopulations from the same species. For the 15 (including *P. serini*) species for which we sampled more than two mites per individual bird, we calculated the genetic diversity of each infrapopulation. The genetic diversity was highly repeatable between infrapopulations of the same species, but differed between species (*R *= 0.79, 95% CI = 0.63–0.94). In addition, we explored if some infrapopulations were biasing estimates of species‐wide genetic diversity. In particular, we tested if there was any difference between the full data set (i.e., five mites per bird) and a partial data set (i.e., one mite per bird) generated via a subsampling simulation (see “Simulation analysis”). There was a significant positive correlation between the average genetic diversity of all infrapopulations from each species and our species genetic diversity values calculated by picking up only one mite from each individual bird (Spearman's correlation, *ρ* = 0.75, *P* < 0.01), thus supporting the robustness of the estimates.

Overall, we are confident that our estimates of genetic diversity at the species level are robust and thus we were able to test for the relationship between the typical infrapopulation size of each mite species (coming from the data set in Díaz‐Real et al. 2014) and our estimate of feather mite genetic diversity at the species level.

#### Neutrality test

Departures from mutation‐drift equilibrium of a Wright‐Fisher model were assessed by calculating Tajima D (Tajima 1989), using the *tajima.test* function of R package PEGAS (Paradis 2010); significance was obtained assuming that D follows a normal distribution with mean zero and variance one. We also investigated departures using the R_2_ test, known to be very powerful in detecting population expansions even for small sample sizes (Ramos‐Onsins and Rozas 2002). R_2_ was calculated using the *R2.test* function implemented in the R package PEGAS (Paradis 2010) and its statistical significance was calculated through 1000 coalescent simulations.

#### Simulations analysis

In order to use all mtDNA sequences available, and to take into account uncertainty on the estimated infrapopulation sizes, we tested the relationship between species infrapopulation size and genetic diversity by running 100 Generalized Linear Models (GLMs). For each run, we recalculated species genetic diversity and infrapopulation size of each species as explained below.

Feather mite infrapopulations are thought to be created mainly by vertical transmission of mites from bird parents to offspring (Proctor 2003). It is thus expected that mites within a bird individual would not be genetically independent (even probably including close relatives). In fact, we found mites from the same bird individual often sharing the same haplotype (Figs. S3–S12 Supporting information), thus potentially underestimating species genetic diversity (see Simulation details). Therefore, we created 100 new data sets, each having a genetic diversity estimate (*π*) for each mite species that was calculated by randomly sampling a single mite individual from each individual bird (see Data, Table S1 Supporting information). We used the data generated in each iteration to test the statistical relationship between genetic diversity and infrapopulation size (see below; “Statistical analysis”). In the same way, we also used this simulation to estimate Tajima's D and R_2_ values for each mite species. The average of the estimates from all iterations was used as the representative estimate of genetic diversity, Tajima's D, or R_2_ values for each species (Table 1).

**Table 1:** Feather mite species and bird host names, number of mites (*n*) and number of infrapopulations (If) sampled, number of polymorphic sites (*S*), and demographic statistics results (*D*,* R*_2_, and *R*_2%_ sig.). Demographic statistic values reported are the average from 100 iterations (see Materials and Methods). The % sig. column shows the percentage of statistically significant iterations. Species with percentages of significant iterations higher than 95% or 75% were considered as significant (**) or marginally significant (*) respectively. Tajima's D only was statistically significant for some iterations in *P. rubeculinus* (33%) and *P. serini* (57%)

Feather mite species	Bird species	*n*	If	*S*	*D*	*R*_2_	*R*_2%_ sig.
*Dolichodectes hispanicus*	*Hippolais polyglotta*	30	10	22	−1.10	0.08	100**
*Monojoubertia microphylla*	*Fringilla coelebs*	35	15	46	−0.93	0.09	79*
*Proctophyllodes acanthicaulus*	*Muscicapa striata*	10	10	26	−1.50	0.09	100**
*Proctophyllodes ateri*	*Periparus ater*	29	9	10	−0.01	0.18	1
*Proctophyllodes cetti*	*Cettia cetti*	29	9	22	−0.29	0.13	21
*Proctophyllodes clavatus*	*Sylvia borin*	14	8	23	−0.41	0.12	52
*Proctophyllodes cotyledon*	*Phoenicurus ochruros*	24	8	11	−1.62	0.13	36
*Proctophyllodes doleophyes*	*Ficedula hypoleuca*	29	9	22	−1.19	0.09	100**
*Proctophyllodes lusciniae*	*Luscinia megarhynchos*	41	21	23	−1.51	0.06	100**
*Proctophyllodes motacillae*	*Motacilla flava*	11	11	43	−1.55	0.07	100**
*Proctophyllodes musicus*	*Turdus merula*	29	9	17	−0.52	0.12	42
*Proctophyllodes rubeculinus*	*Erithacus rubecula*	37	17	10	−1.88	0.08	76*
*Proctophyllodes schoenicli*	*Emberiza schoeniclus*	9	9	18	−1.80	0.07	100**
*Proctophyllodes serini*	*Serinus serinus*	34	14	22	−1.97	0.10	46
*Proctophyllodes stylifer*	*Parus major*	19	10	25	−0.67	0.09	100**
*Proctophyllodes sylviae*	*Sylvia atricapilla*	32	12	59	−0.71	0.09	85*
*Pteronyssoides parinus*	*Cyanistes caeruleus*	28	8	19	0.25	0.16	4
*Trouessartia rosterii*	*Sturnus unicolor*	12	6	21	−1.19	0.12	57

We also used this simulation approach to explore the robustness of our results against uncertainty in infrapopulation size per mite species. The feather mite loads in Díaz‐Real et al. (2014) are based on counts of all feather mites living on the wing of each bird host (i.e., counting all mites without differentiating mite species). It should be noted that two or three feather mite species often inhabit the wing of a same bird species (Proctor 2003). However, we studied the genetic diversity of the most abundant mite species occurring on each bird species (see above). Therefore, the median infrapopulation size reported by Díaz‐Real et al. (2014) is either the real median of our focal mite species (if this was the only species occurring in the bird host) or a slightly overestimated value (i.e., if other species were in that bird). Therefore, to consider this uncertainty, for each of the 100 iterations (see above) we also calculated a new median infrapopulation size value for each mite species by extracting a pseudo‐random value between half and the real median load of feather mites per bird species reported in Díaz‐Real et al. (2014). Nevertheless, a similar positive relationship with the genetic diversity (see Results) was obtained when using the non‐simulated infrapopulation size data (GLM: *F *= 9.00; df = 15, *P *< 0.01).

#### Statistical analysis

We used the most comprehensive phylogeny for European plumicolous feather mite species (Doña et al. 2015) to control for phylogenetic non‐independence of the data (Felsenstein 1985; Freckleton et al. 2002). We calculated the phylogenetic signal for genetic diversity estimating the maximum likelihood value of Pagel's *λ* (Pagel 1999). We used the function *pgls* of the R package Caper (Orme 2012) version 0.5.2 (http://caper.r-forge.r-project.org/). Mean *λ* was < 0.001 for genetic diversity (Fig. S1), not significantly different from 0 (*P*=1), but significantly different from 1 (P<0.001). Genetic diversity was always the response variable in our analysis. Thus, as response variables are the most influent on residuals, it is unlikely that our residuals could show phylogenetic signal.

Therefore, we used a non‐phylogenetic approach (Revell 2010): we analysed the relationship between genetic diversity and infrapopulation size using GLMs with a Gaussian distribution of errors and an identity link function. We performed one GLM for each simulation iteration (see above, Table S2. Supporting information). We used the *glm* function of the STATS R package (R Development Core Team, 2013). The genetic diversity of feather mite species was the dependent variable and the median infrapopulation size was the independent variable. Sample size (number of analysed mites per bird species) varied among mite species, but it was uncorrelated with their genetic diversity (Spearman's correlation, *ρ *= −0.31, *P *= 0.1962). Even so, we used the “weights” option of the *glm* function to give more confidence to data coming from higher sample sizes. We confirmed assumptions underlying GLMs by exploring regression residuals for normality against a Q‐Q plot.

## Results

Non‐significant departures from mutation‐drift equilibrium were found for Tajima's D, but 7 out of 18 (including *P. serini*) species displayed positive and significant low values in all iterations for the most conservative R_2_ statistic (a signature of population expansion).Three other species showed the same patterns but only with marginal statistical significance (Table 1). Despite so, species presented high and contrasting genetic diversity values (Table 1).

**Figure 2:**
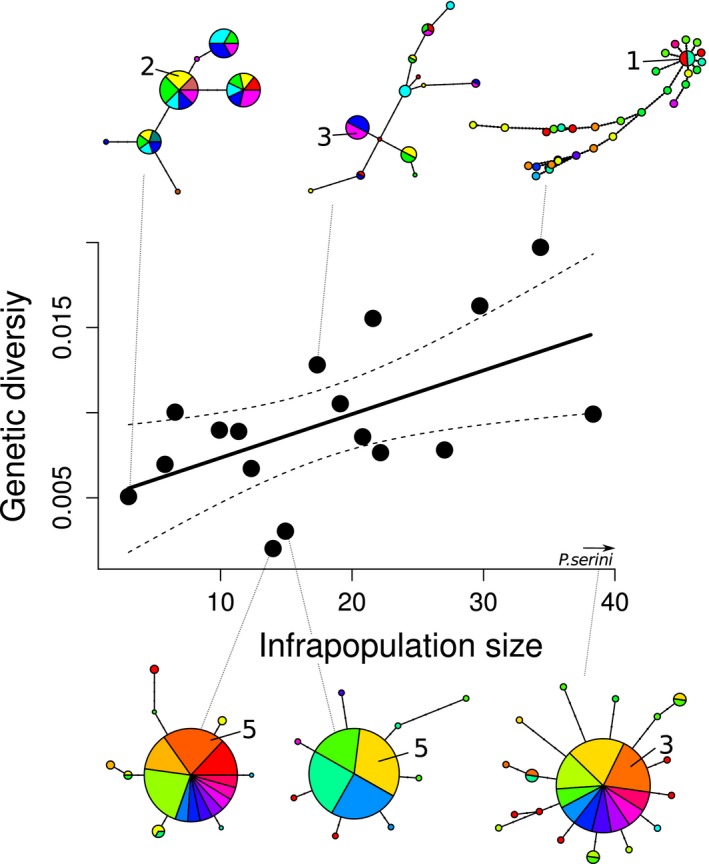
Relationship between infrapopulation size and nucleotide diversity (π) across feather mite species. Haplotype network examples, from top left to right: *Proctophyllodes ateri, P. cetti and P. sylviae*; bottom left to right: *P. rubeculinus, P. cotyledon and P. serini*. Each color in pie charts represents an individual bird. Numbers indicate for each network the maximum number of mites sharing a given haplotype into an individual bird. *P. serini* infrapopulation size was 49, and its genetic diversity 0.003.

When excluding *P. serini*, we found a strong positive relationship between infrapopulation size and genetic diversity across species, and results were robust across the 100 GLMs performed: *F* mean (95%CI) = 8.66 (7.61–9.71), df = 15, P mean (95% CI) = 0.03 (0.02–0.04), explaining a mean (95% CI) of 34% (31–36%) of the original deviance. Similarly, the relationship between the median infrapopulation size and the mean *π* calculated for each species across the 100 iterations (see “Simulation analysis”) explained 37% of the original deviance (GLM: *F *= 8.98; df = 15, *P *< 0.01; Fig. 2).

Despite being the species with the largest median infrapopulation size, *P. serini* showed, as predicted (see Materials and Methods and Discussion), an extremely low genetic diversity, and a star‐like haplotype network. Including this species into the analyses lead to a statistically non‐significant relationship between median infrapopulation size and mean genetic diversity (GLM: *F* = 0.74; d.f. = 16, *P* = 0.40). Interestingly, not only this species, but also *P. rubiculinus* and *P. cotyledon* showed low values of genetic diversity according to their infrapopulation sizes, and the three species shared a strong star‐like haplotype network structure, in contrast with the more reticulated haplotype network structure of the other species (Figs. 2; S2–S12, Supporting information). The original deviance explained by the model increased to 42% when excluding these three star‐like species from the analysis (GLM: *F *= 9.27; df = 15, *P *< 0.01).

## Discussion

Our results show a strong positive relationship between median infrapopulation size and genetic diversity across 17 feather mite species. Therefore, our findings are in line with previous preliminary evidence suggesting that a simple ecological measure such as the median number of parasites per infected host individual is a relevant variable to understand evolutionary processes at the species level (Criscione et al. 2005). So, disentangling why symbionts such as feather mites differ so strongly in their infrapopulation sizes among species (Díaz‐Real et al. 2014) is a necessary ingredient to understand the evolutionary ecology of these host‐symbiont systems. In fact, while species richness has been a major topic in the study of the evolutionary ecology of parasites, understanding infrapopulation size variation between parasite species and parasite load between host species is still a major challenge (Poulin and George‐Nascimento 2007).

In spite of the importance of our results, there is still a considerable amount of variance in mtDNA diversity that remains unexplained by median infection intensities. There are several possible and not necessarily mutually exclusive explanations for this. First, departures from mutation‐drift equilibrium can distort the median infrapopulation size ~ genetic diversity relationship (Crow and Kimura 1970). That is, historical demographic events (expansions and bottlenecks) may distort levels of diversity beyond what one might expect based on a strict census to effective size relationship. For example, Dabert et al. (2015) reported low genetic diversity for another feather mite species, *Zachvatkinia isolate*. They interpreted this as a consequence of bottlenecks and founder effects occurring during bird‐to‐bird feather mite transmission. Interestingly, our data reveal several signatures of population expansions based on the R_2_ tests (Table 1) that may have introduced noise into the data set. For instance, the low genetic diversity values coupled with the shapes of the networks for *P. rubiculinus* and *P. cotyledon* suggested extreme recent demographic changes or a selective sweep (Shaw 2002; Bensch et al. 2006; Irwin et al. 2009; Rato et al. 2011 **)**. In effect, if we exclude these two species from the analysis the model fitted the data better (the original deviance explained increases to 42%), providing further evidence of the importance of the demographic and evolutionary context on the relationship between genetic diversity and population size.

Species divergence history may also be important in influencing an infrapopulation size ‐ genetic diversity relationship. Regardless of the infrapopulation sizes of the species, recently diverged species may show low genetic diversity because they would have retained only a fraction of mutations that were segregating in the parental species (Barton and Charlesworth 1984; Klicka and Zink 1997; Nichols 2001; De Brito et al. 2005). As an extreme example of this, we highlight the effect of *P. serini*. This species is part of the Pinnatus group. The *Pinnatus* group of the genus *Proctophyllodes* is the best known example of this sort of processes among feather mites from European passerines. This is a group identified by taxonomists as being constituted by close morphological species (Atyeo and Braasch 1966; Badek et al. 2008), and a multilocus phylogenetic study showed that this is a recent species complex with rapid diversification rate (Knowles and Klimov 2011). In addition, we recently found that COI barcoding was unable to delimitate between morphological species within this complex (Doña et al. 2015). Here, we have studied one of these species, *P. serini*, living on the feathers of European serins (*Serinus serinus*), and we found an extremely low genetic diversity (Goodall‐Copestake et al. 2012), regardless of its large infrapopulation sizes (see Fig. 2).

Another factor introducing noise on the correlation between mtDNA genetic diversity and infrapopulation size could be that infrapopulation size is not an accurate reflection of the total census size (component population) of feather mites. Further work is needed here. Lastly, the high genetic diversity values found for most species (Goodall‐Copestake et al. 2012) could be explained by their high mutation rate (Hodgkinson and Eyre‐Walker 2011), as reported in previous feather mite studies (Dabert et al. 2010; Štefka et al. 2011).

Overall, our study shows that infrapopulation size does indeed explain a significant amount of the among‐species variation in genetic diversity of parasites. Infrapopulation size is easier to estimate than census size or *N*_*e*_. In addition, the relationship between infrapopulation size and genetic diversity we report here supports the idea that ecological processes occurring at the within‐individual host scale are key to understand the distribution of genetic variation among parasite species. However, how other factors such as prevalence, host census size, or other measures of infrapopulation size variation (such as the harmonic mean) may influence the genetic diversity of feather mites will require further studies. Moreover, we report that pervasive demographic processes in host‐parasite systems such as bottlenecks or population growth events could strongly impact the species genetic diversity. This should encourage further research of underlying mechanisms of infrapopulation dynamics that will be relevant to achieve a complete picture of the factors shaping parasite species genetic diversity.

## Conflict of Interest

None declared.

## Data Accessibility

DNA sequences: GenBank accessions: KT025257‐KT025578.

Sequence alignment and simulation results are deposited in Dryad: doi:10.5061/dryad.93sh8. The R code used for simulation is available on the GitHub repository: https://github.com/Jorge-Dona/Infrapopulation_genetics.

## Supporting information


**Figure S1.** Log likelihood profile of lambda estimation (here is shown only one iteration calculation.
**Figure S2–S13.** Each colour in pie charts represents an individual bird. Pie size represents haplotype frequency.

**Table S1**. Sampling details: locality, date of sampling, sample size, samples id, collectors and GenBank accesions.
