# Supplementary material for: Species mtDNA genetic diversity explained by infrapopulation size in a host‐symbiont system
Source: Ecol Evol. 2015 Nov 24;5(24):5801–9. doi: 10.1002/ece3.1842 (PMC4717341; doi:10.1002/ece3.1842)
Supplement: Supplementary file 1 — Figure S1. Log likelihood profile of lambda estimation (here is shown only one iteration calculation. Figure S2–S13. Each colour in pie charts represents an individual bird. Pie size represents haplotype frequency. [file ECE3-5-5801-s001.pdf]

Figure.S1

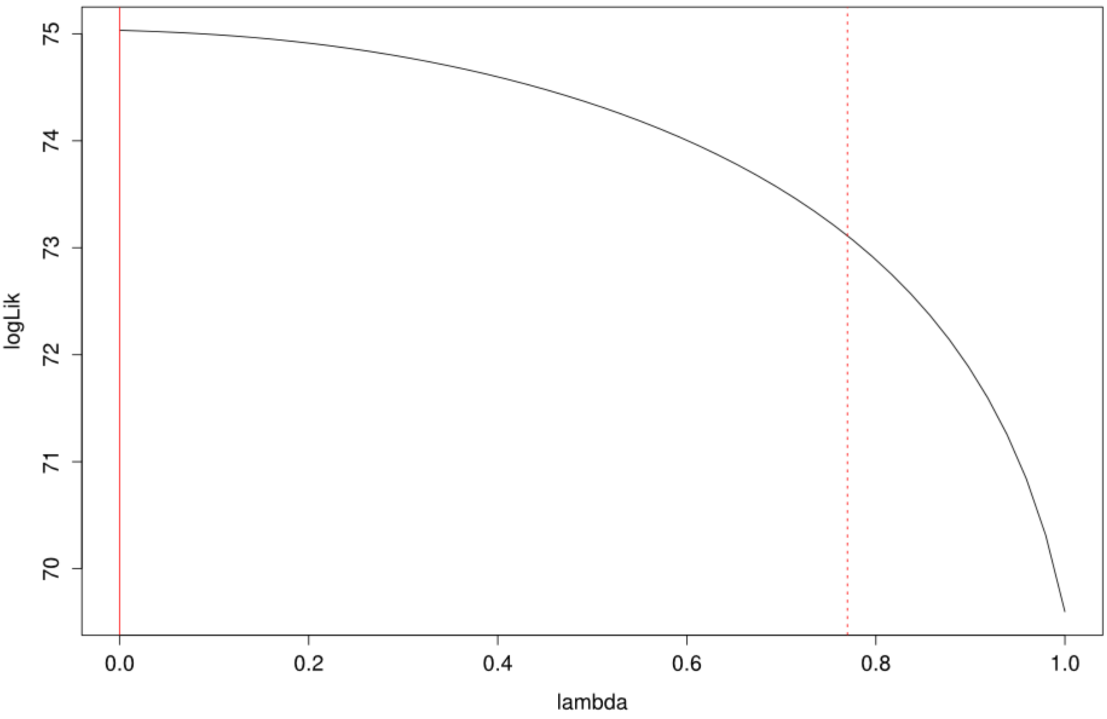

Log likelihood profile of lambda estimation (here is shown only one iteration calculation).

Figure S2-13. Each colour in pie charts represents an individual bird. Pie size represents haplotype frequency.

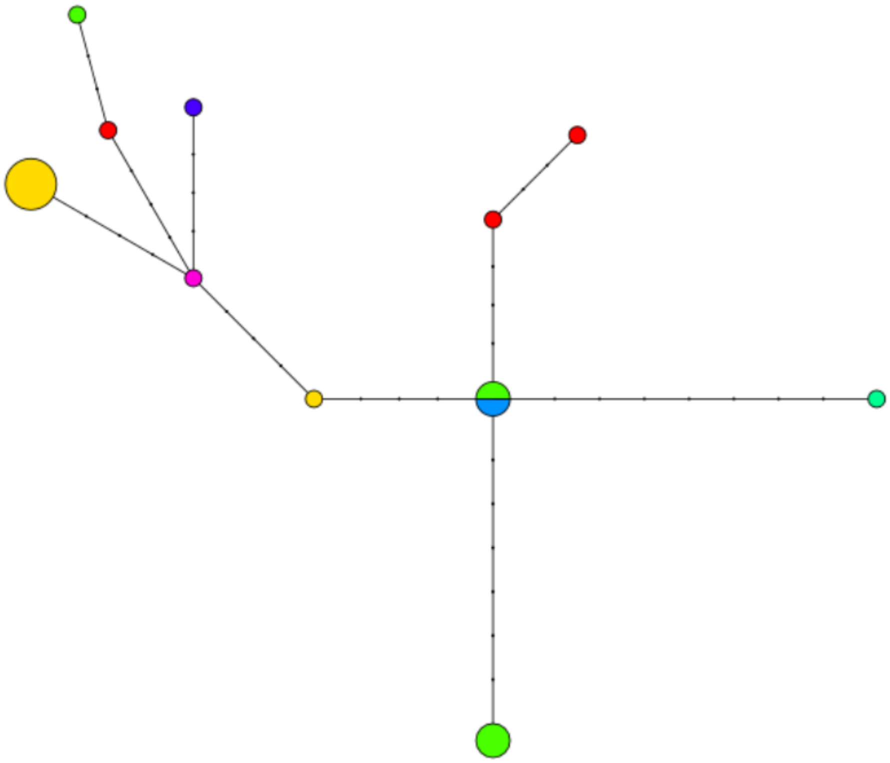

Proctophyllodes clavatus haplotype network.

Figure.S3

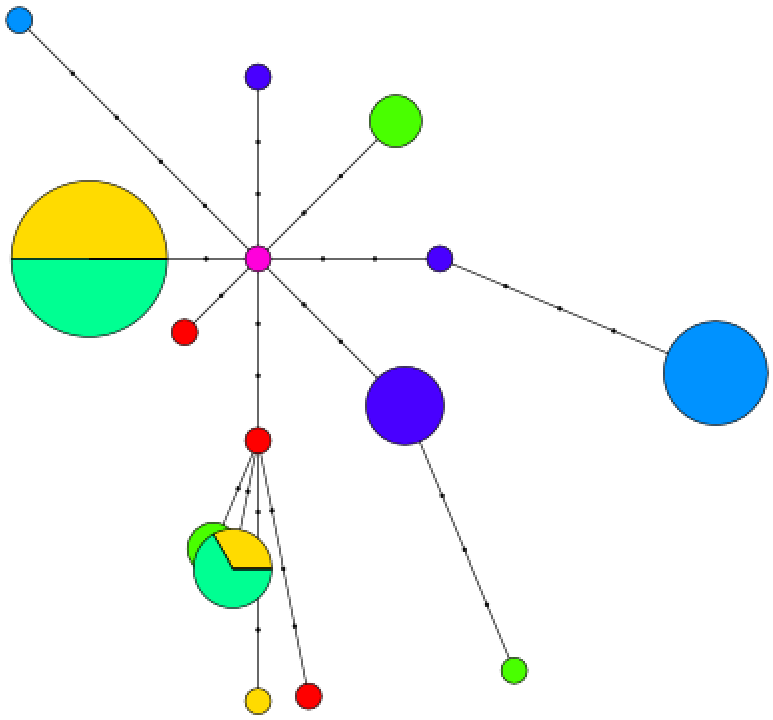

Proctophyllodes doleophyes haplotype network.

Figure.S4

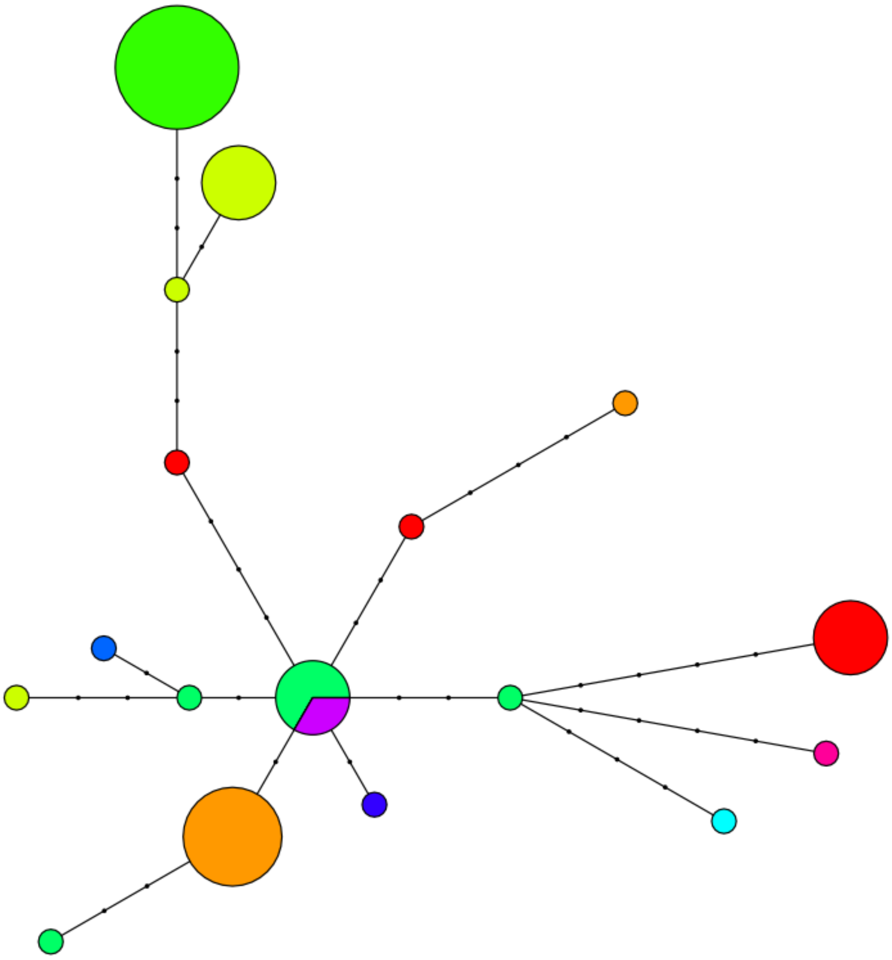

Dolichodectes hispanicus haplotype network.

Figure.S5

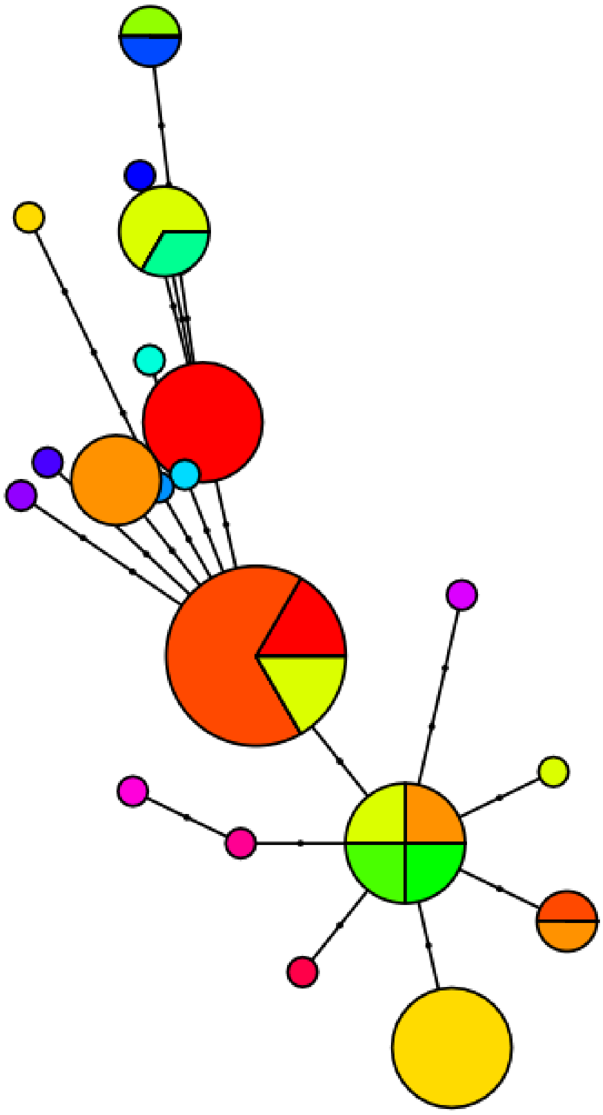

Proctophyllodes lusciniae haplotype network.

Figure.S6

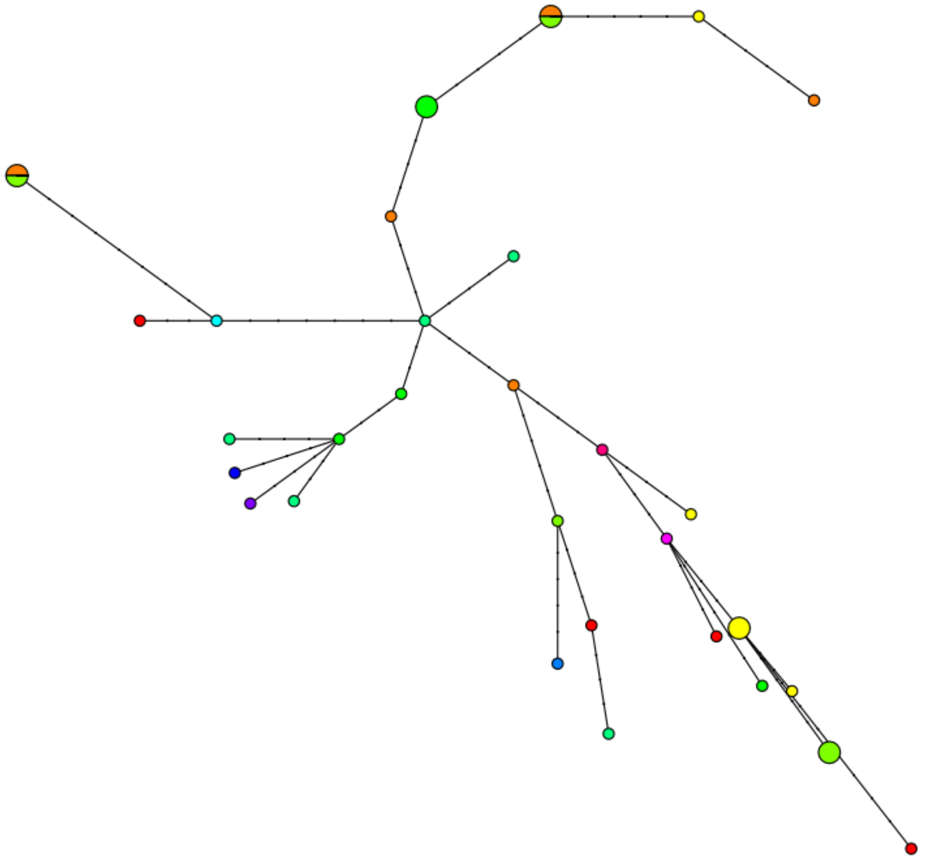

Monojoubertia microphylla haplotype network.

Figure.S7

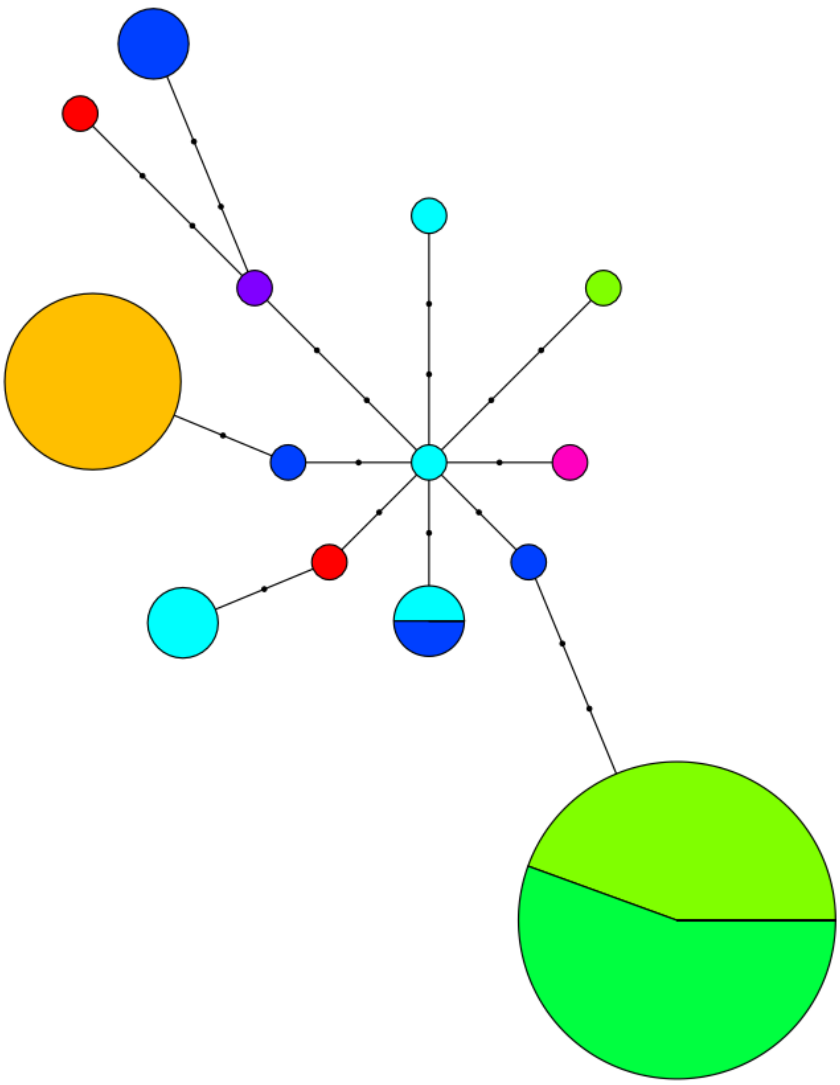

Proctophyllodes musicus haplotype network.

Figure.S8

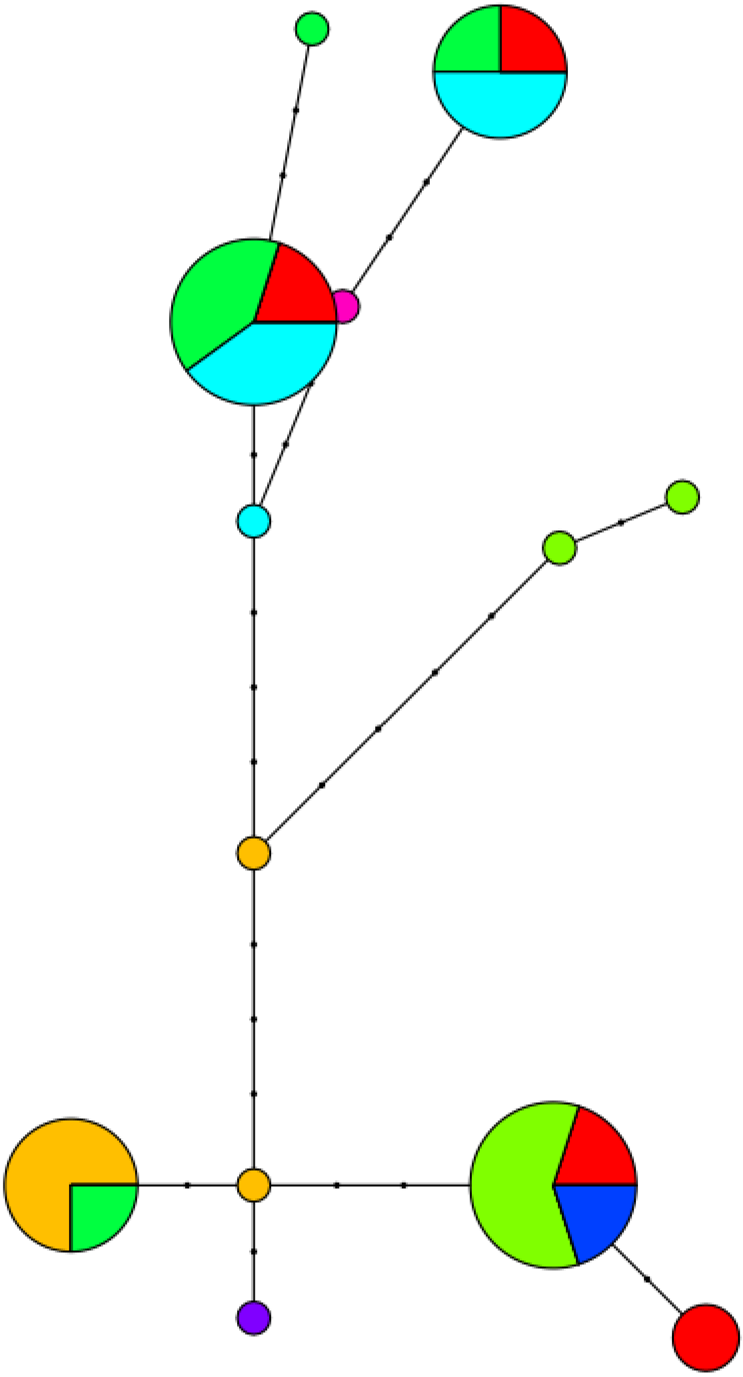

Pteronyssoides parinus haplotype network.

Figure.S9

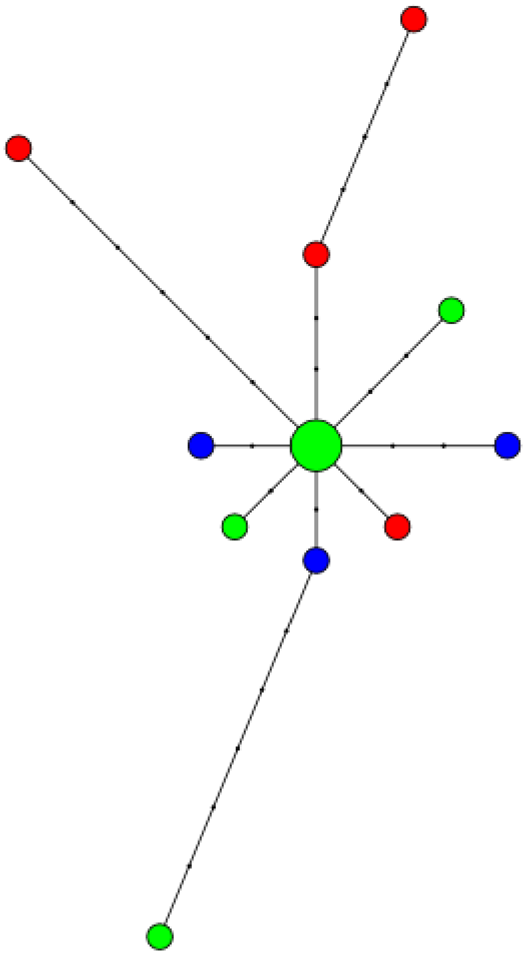

Trouessartia rosterii haplotype network.

Figure.S10

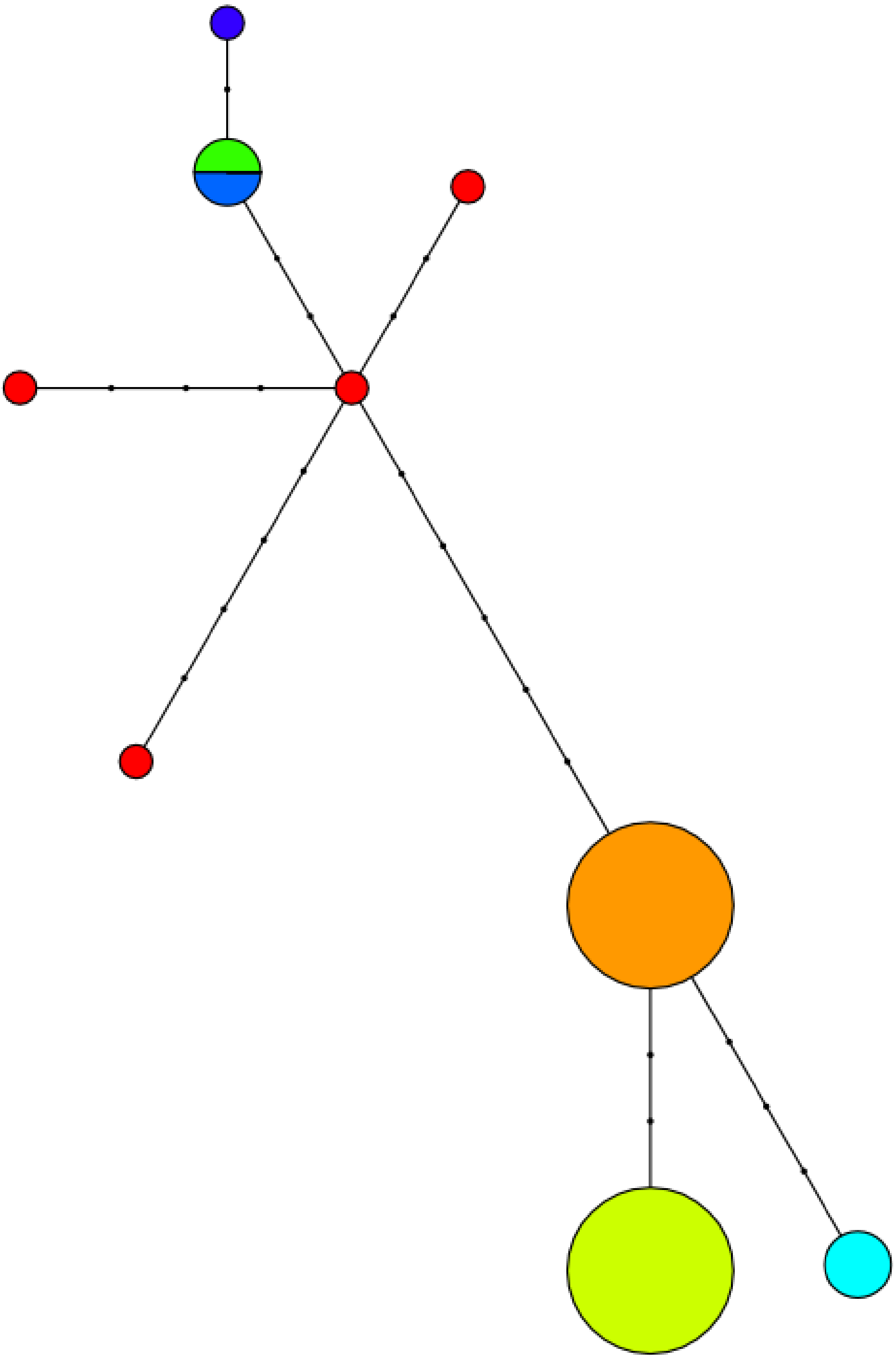

Proctophyllodes stylifer haplotype network.

Figure.S11

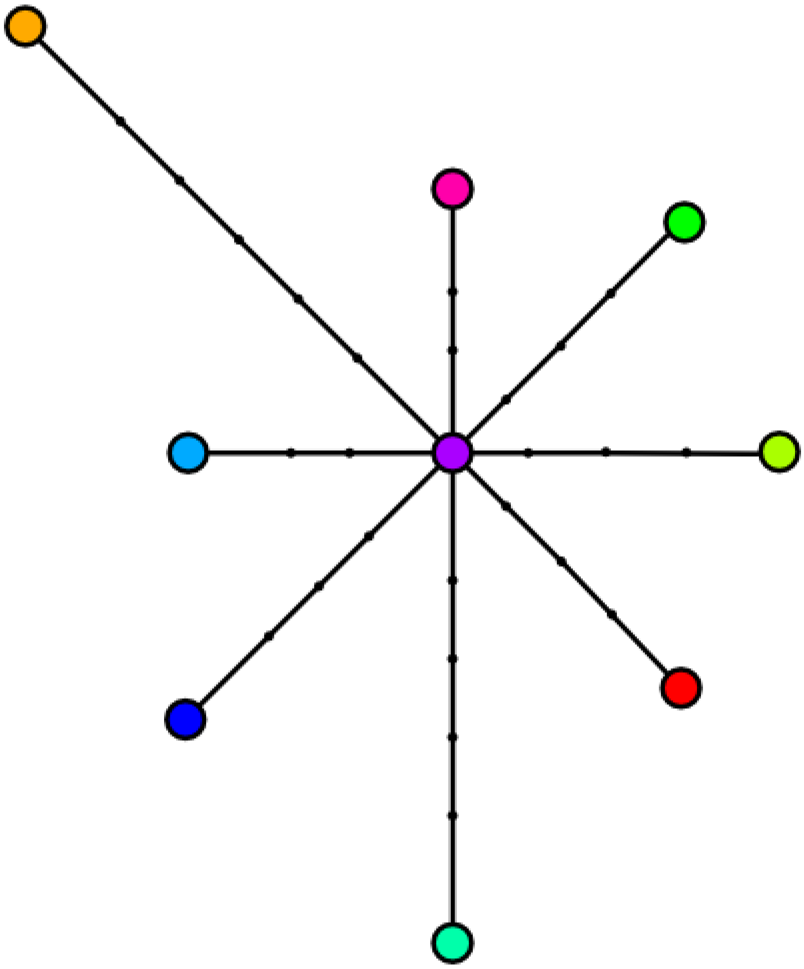

Proctophyllodes schoenicli haplotype network.

Figure.S12

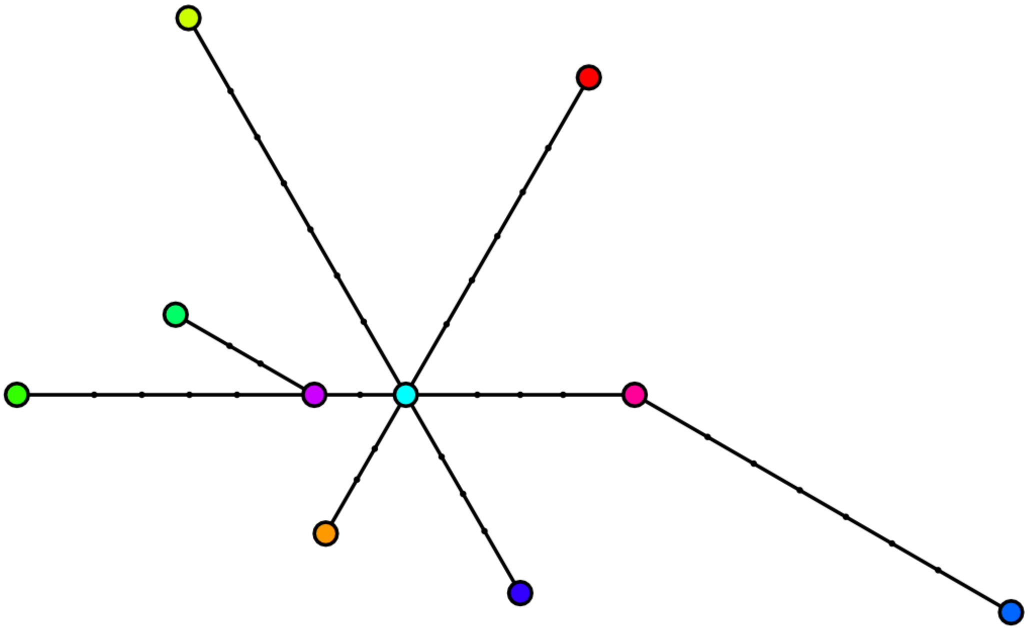

Proctophyllodes acanthicaulus haplotype network.

Figure.S13

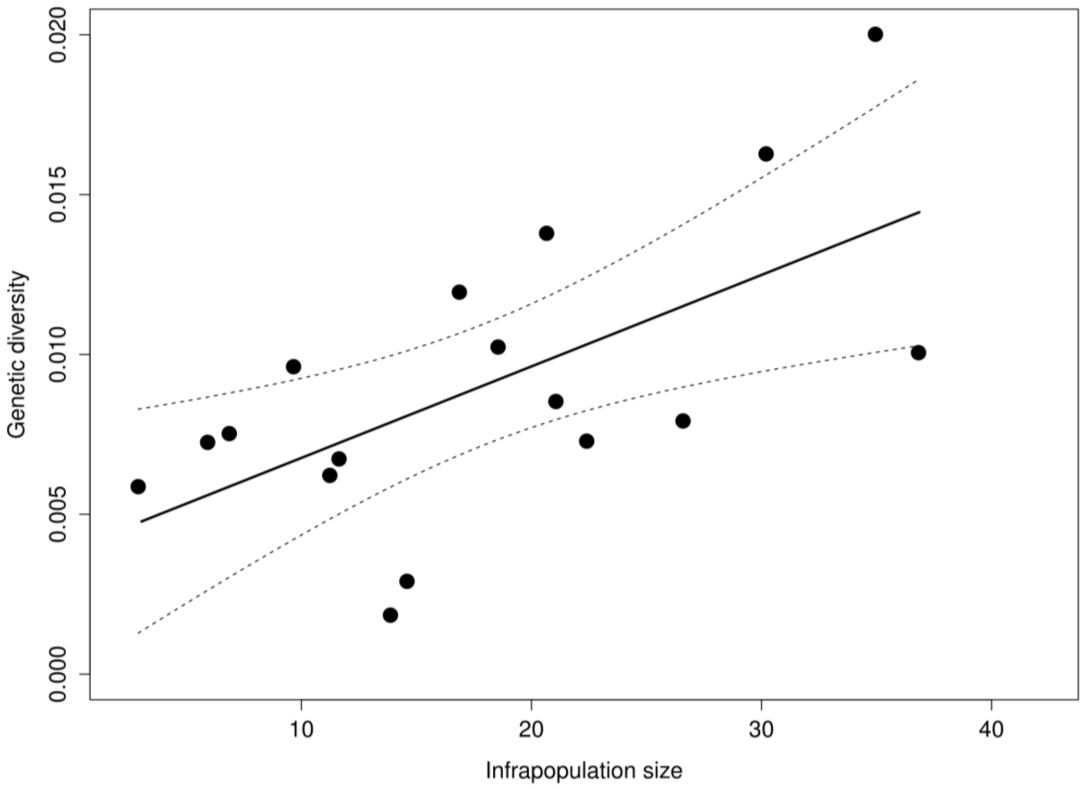

Scatter plot with GLM regression line showing the relationship between infrapopulation size and genetic diversity of each feather mite species excluding Russian samples.
